# Impact of the COVID-19 pandemic on CHD care and emotional wellbeing

**DOI:** 10.1017/S1047951120004758

**Published:** 2020-12-14

**Authors:** Melissa K. Cousino, Sara K. Pasquali, Jennifer C. Romano, Mark D. Norris, Sunkyung Yu, Garrett Reichle, Ray Lowery, Suzanne Viers, Kurt R. Schumacher

**Affiliations:** 1 Department of Pediatrics, Michigan Medicine, Ann Arbor, MI, USA; 2 University of Michigan Congenital Heart Center, C.S. Mott Children’s Hospital, Ann Arbor, MI, USA; 3 Department of Cardiac Surgery, Michigan Medicine, Ann Arbor, MI, USA

**Keywords:** COVID-19, heart disease, transplant, cardiomyopathy, stress

## Abstract

**Background:**

Understanding the impact of the COVID-19 pandemic on paediatric non-COVID-19-related care, as well as patient and caregiver concerns and stressors, is critical for informing healthcare delivery. It was hypothesised that high care disruptions and psychological stress would be observed among paediatric and adult CHD patients in the early phase of the pandemic.

**Methods:**

A cross-sectional, international, electronic survey study was completed. Eligible participants included parents of children with acquired or CHD, adults with CHD, or caregivers of adults with CHD.

**Results:**

A total of 1220 participants from 25 countries completed the survey from 16 April to 4 May, 2020. Cardiac care disruption was significant with 38% reporting delays in pre-pandemic scheduled cardiac surgeries and 46% experiencing postponed cardiac clinic visits. The majority of respondents (75%) endorsed moderate to high concern about the patient with heart disease becoming ill from COVID-19. Worry about returning for in-person care was significantly greater than worry of harm to patient due to postponed care. Clinically significant psychological stress was high across the sample including children (50%), adults with CHD (42%), and caregivers (42%).

**Conclusions:**

The early phase of the COVID-19 pandemic contributed to considerable disruptions in cardiac care for patients with paediatric and adult CHD. COVID-19-related fears are notable with potential to impact willingness to return to in-person care. Psychological stress is also very high necessitating intervention. Further study of the impact of delays in care on clinical outcomes is warranted.

As severe acute respiratory syndrome coronavirus 2 (SARS-COV-2) rapidly spread throughout the world and United States of America, governments and hospital systems responded to calls for physical distancing, personal protection equipment preservation, and preparations for a surge in COVID-19 patients. Out of concern for vulnerable patients and healthcare workers, in-person healthcare was drastically reduced and elective surgeries and diagnostic procedures postponed during the early phase of the pandemic.^1^ The impact of these changes in our healthcare delivery system for patients with acute and chronic conditions unrelated to COVID-19, like paediatric and adult CHD, is not well understood.^2^

CHDs are the most common birth defects, with >40,000 congenital cardiac operations performed each year in the Unites States of America,^3^ and >11 million people living with CHD globally.^4^ While this population at large is likely not at risk for significant COVID-19-related illness;^5,6^ they are highly susceptible to the indirect effects of changes and delays in care delivery related to the pandemic. In addition, this population has significant mental health comorbidities at baseline,^7–9^ which additional stressors related to the pandemic could exacerbate. Understanding the extent to which the pandemic has impacted care delivery and stress within the paediatric and adult CHD population can better inform our response and design of care during the course of the pandemic and amidst surges in cases.

We conducted an international online survey aiming to: 1) describe disruptions in cardiac care among paediatric and adult patients with CHD, 2) assess COVID-19-related and general psychological stress, 3) examine disease- and medical care-related correlates of psychological stress, and 4) determine support needs of patients and their caregivers during the pandemic. It was hypothesised that high care disruptions and psychological stress would be observed among paediatric and adult CHD patients in the early phase of the pandemic.

## Materials and method

### Study design and measures

An electronic survey measuring impact of COVID-19 on cardiac care and related concerns was administered. This survey was developed by an expert, multidisciplinary panel with experience in survey design, psychology, and clinical expertise in multiple domains of cardiac and surgical care. Two parents also reviewed the survey and offered feedback. The survey included fixed response and Likert rating scale items across the following domains: demographic/disease information, COVID-19-related cardiac care disruptions, health concerns, information sources, emotional/behavioural stressors, and support needs (see Online Supplement for survey instrument). In addition, the eight-item parent-reported NIH PROMIS Psychological Stress Experiences Scale^10^ and 10-item self-reported NIH Toolbox Perceived Stress Scale^11,12^ were administered as validated measures of general life stress. No direct patient identifying information was included. The survey bundle, which took <10 minutes to complete, was administered using Qualtrics survey software (Provo, UT) with dynamic routing capabilities. Some questions could be skipped allowing participants to the survey without answering questions they did not know or understand. The study was reviewed and approved by the University of Michigan Institutional Review Board with electronic consent obtained from participants.

### Participants and recruitment

Eligible participants included 1) a parent of a child or young adult (0–25 years) with CHD, cardiomyopathy or heart transplant, 2) an adult with CHD (18+ years), or 3) a partner or spouse of an adult with CHD who provided caregiving support for >1 year. Participants were recruited through a social media campaign with at least 11 paediatric and adult CHD organisations advertising the survey (see Acknowledgements for participating organisations). This methodology for recruiting patients and obtaining information from individuals with congenital and other rare heart disease has been previously utilised and found to provide valid information by our group.^13,14^ The survey was also sent via e-mail to patients at our centre who had previously undergone congenital heart surgery and participated in a programme for longitudinal follow-up as previously described.^15^ The survey was open during the early phase of the pandemic for a 3-week period from 16 April to 4 May, 2020.

### Statistical analysis

Data are reported as frequency with percentage for categorical variables and mean ± standard deviation (SD) or median with interquartile range for continuous variables. A five-point Likert scale was used for each item on concerns specific to COVID-19 and healthcare, ranging from “not concerned” to “very concerned,” and was linearly transformed to a 0 to 100-point scale with higher scores indicating more concern. General psychological stress was evaluated by T-scores generated from self-reported NIH Toolbox Perceived Stress Scale^12^ and parent-reported NIH PROMIS Psychological Stress Experiences Scale,^10^ with higher scores indicating more stress. A within-respondent comparison between COVID-19-related healthcare concerns was made using paired t-test. Univariate associations of demographic and disease-specific characteristics with COVID-19-related health concerns and psychological stress were examined using two-sample t-test or analysis of variance, as appropriate. All analyses were performed using SAS version 9.4 (SAS Institute, Cary, NC, USA), with a statistical significance level of 0.05 using two-sided tests.

## Results

### Participant characteristics

Participant characteristics are reported in Table 1. Of the 1220 respondents, the majority were parent/caregivers of children (65%); however, a considerable sample of adult CHD patients participated (n = 352, 29%). Country of origin was primarily the United States of America (93%), yet, 25 countries were represented by the data including 24 participants from the United Kingdom and 23 participants from Canada. The participants included a high proportion of patients with critical, complex CHD with 48% having single-ventricle anatomy and most status post staged Fontan palliation (65% of single-ventricle participants). Participants were primarily receiving outpatient cardiac care at time of survey completion (96%); however, prior interventions and hospitalisations were notable with 76% of the respondents having a history of two or more cardiac surgeries.

**Table 1.** Respondent characteristics.*

Respondent	
Patient	352 (28.9%)
Parent/caregiver	858 (70.3%)
Partner/spouse	9 (0.7%)
Not reported	1 (0.1%)
Patient age, years	
(n = 1219)	10 (4–28)
< 18 years	793 (65.0%)
≥ 18 years	426 (34.9%)
Not reported	1 (0.1%)
Country	
United States of America	1134 (93.0%)
Northeast	220/1134 (19.4%)
Midwest	429/1134 (37.8%)
South	240/1134 (21.2%)
West	243/1134 (21.4%)
Non-United States of America	77 (6.3%)
Canada	23 (1.9%)
United Kingdom	24 (2.0%)
Not reported	9 (0.7%)
Heart disease	
Single-ventricle CHD	581 (47.6%)
Two-ventricle CHD	437 (35.8%)
Cardiomyopathy or disease of the heart muscle without structural heart disease	62 (5.1%)
Status post-heart transplant	52 (4.3%)
Not reported	88 (7.2%)

* Data are presented as n (%) for categorical variables and median (interquartile range) for continuous variable.

### COVID-19 impact on cardiac care

Table 2 provides information on the impact of COVID-19 on cardiac care. Of the 141 (12%) participants with a recent or upcoming cardiac surgery planned, 38% of this subgroup experienced postponement of surgery. Many others expressed worry about the impact of COVID-19 on their still scheduled surgery (62%). Similar rates of postponed cardiac catheterisations were reported in 150 participants with 40% of this subgroup already experiencing delays. Nearly, half of the participants (46%) had a cardiology clinic visit postponed due to the pandemic. Only a quarter of respondents had their in-person clinic visit changed to a video visit or telephone call; however, 68% of those who did have a telemedicine visit reported it adequately met their needs.

**Table 2.** COVID-19 impact on cardiac care (n = 1220).*

Recent or upcoming surgery planned	141 (11.6%)
Surgery postponed due to the COVID-19 pandemic	54/141 (38.3%)
Worried about impact of pandemic on a scheduled surgery that has not yet been postponed (n = 87)	54/87 (62.1%)
Recent or upcoming cardiac catheterisation planned	150 (12.3%)
Cardiac catheterisation postponed due to the COVID-19 pandemic	60/150 (40.0%)
Worried about the impact of pandemic on a scheduled catheterisation that has not yet been postponed (n = 90)	62/90 (41.3%)
Recent or upcoming cardiology clinic appointment planned	894 (73.3%)
Clinic appointment postponed due to the COVID-19 pandemic	414/894 (46.3%)
Worried about the impact of pandemic on a scheduled clinic appointment that has not yet been postponed (n = 480)	360/480 (75.0%)
Received information about rescheduling the appointment until after the pandemic	300/894 (33.6%)
Appointment changed from in person to a video visit or phone call due to pandemic	314 (25.7%)
Felt the virtual visit was adequate to cover the needs	214/314 (68.2%)

* Data are presented as n (%).

### COVID-19 information sources

The majority of participants (66%) received information about COVID-19 from a heart disease support, advocacy, or social media group. Many viewed articles (65%) and webinars (54%). A subset (40%) received COVID-19 information directly from their cardiologist/surgeon/heart centre, with few receiving information specific to them or their condition from their care team or centre (21%).

### Concerns related to COVID-19 and cardiac care

Participants endorsed a number of concerns specific to COVID-19 and their cardiac care or health (Table 3). The greatest concern was a child or partner with heart disease becoming ill from the disease with 75% being moderately to very concerned. Personally becoming ill with COVID-19 (parent/caregiver or adult patient) was also of high concern in more than half of the sample (54%) with many worrying a great deal about who would care for the child or adult with heart disease if infected (54%). Notably, worry about coming to the hospital or clinic for in-person care (mean 60.4 ± SD 34.4) was significantly greater than worry of harm to patient due to postponed medical care (mean 29.0 ± SD 33.9; p < 0.0001). Compared to the 75% worried about COVID-19 infection and illness, only a quarter (27%) of respondents expressed considerable worry about not being seen in person by their cardiology provider due to the pandemic in spite of the large proportion of severe CHD. Death of a patient due to postponed medical care was of moderate to high concern among 12% of the participants.

**Table 3.** Concerns related to COVID-19 and cardiac care (n = 1220).*

	Overall score^†^	Not concerned	A little concerned	Somewhat concerned	Moderately concerned	Very concerned	Not reported
Concerns related to COVID-19							
Child/partner becoming ill from COVID-19 (n = 867)	80.9 ± 25.6	12 (1.4)	62 (7.2)	104 (12.0)	194 (22.4)	459 (52.9)	36 (4.2)
Personally becoming ill from COVID-19	65.6 ± 30.6	57 (4.7)	191 (15.7)	257 (21.1)	290 (23.8)	373 (30.6)	52 (4.3)
Who will take care of the patient if you get sick	64.0 ± 37.1	164 (13.4)	163 (13.4)	190 (15.6)	165 (13.5)	493 (40.4)	45 (3.7)
Impact of COVID-19 on personal finances	52.6 ± 35.0	187 (15.3)	256 (21.0)	260 (21.3)	193 (15.8)	279 (22.9)	45 (3.7)
Heart care concerns							
Obtaining medications during the pandemic	28.6 ± 30.8	474 (38.9)	321 (26.3)	196 (16.1)	102 (8.4)	81 (6.6)	46 (3.8)
Not being seen in person by cardiology provider	43.3 ± 31.4	231 (18.9)	302 (24.8)	311 (25.5)	202 (16.6)	125 (10.2)	49 (4.0)
Not being able to speak to or reach my cardiology provider	27.5 ± 31.5	521 (42.7)	283 (23.2)	174 (14.3)	111 (9.1)	81 (6.6)	50 (4.1)
Postponed surgery or procedure	18.6 ± 30.4	754 (61.8)	161 (13.2)	88 (7.2)	87 (7.1)	66 (5.4)	64 (5.2)
Postponed clinic visit	35.5 ± 31.5	354 (29.0)	299 (24.5)	264 (21.6)	165 (13.5)	83 (6.8)	55 (4.5)
Challenges rescheduling surgery or procedure	20.6 ± 31.8	719 (58.9)	167 (13.7)	99 (8.1)	87 (7.1)	81 (6.6)	67 (5.5)
Harm to patient due to postponed medical care	29.0 ± 33.9	532 (43.6)	258 (21.1)	144 (11.8)	113 (9.3)	116 (9.5)	57 (4.7)
Death of patient due to postponed medical care	18.4 ± 30.7	756 (62.0)	180 (14.8)	81 (6.6)	56 (4.6)	85 (7.0)	62 (5.1)
Coming to hospital or clinic for in-person care	60.4 ± 34.4	142 (11.6)	187 (15.3)	223 (18.3)	263 (21.6)	346 (28.4)	59 (4.8)

* Data are presented as mean ± standard deviation or n (%), as appropriate.

† A five-point Likert scale (ranging from “not concerned” to “very concerned”) of each questionnaire was linearly transformed to a 0- to 100-point scale and averaged, with higher scores indicating more concern.

**Figure 1.**
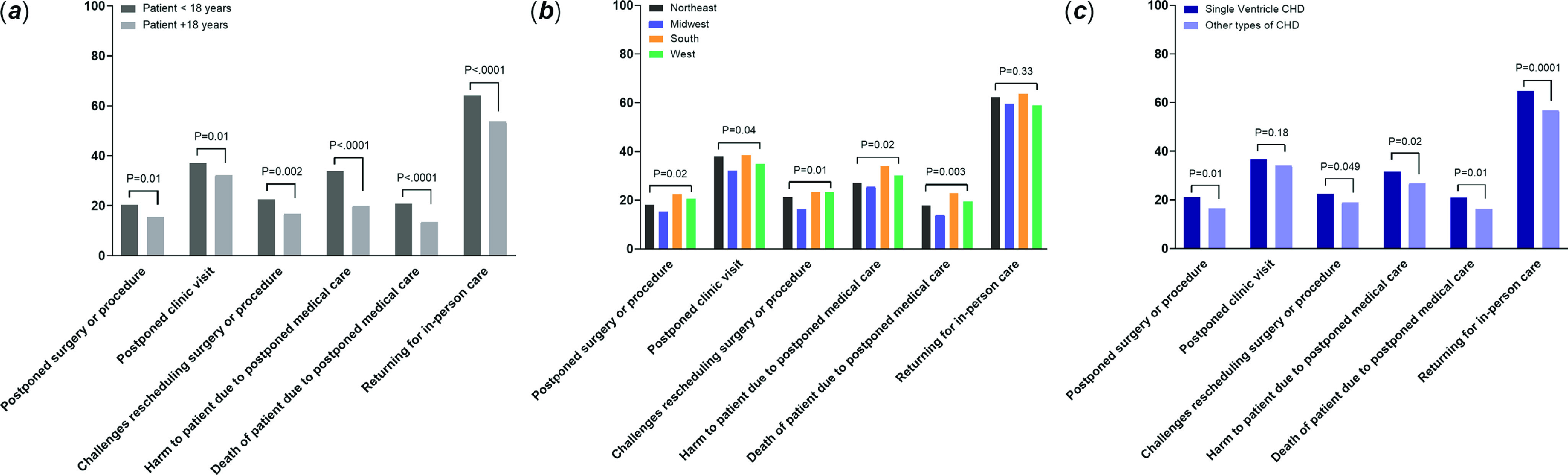
COVID-19-related concerns by patient age, region, and disease type.

Demographic and disease-specific predictors of COVID-19-related health concerns were examined (Fig 1). Parents of children reported significantly higher worry than adult CHD patients or adult caregivers in the following areas: postponed surgery or procedure, postponed clinic visit, challenges rescheduling surgery or procedure, harm to the patient due to postponed care, death of the patient due to postponed care, and coming to the hospital or clinic for in-person care. There were also observed differences in COVID-19-related health concerns by United States region, with higher worries being reported in the United States Northeast and South regions. COVID-19-related health concerns were also significantly greater among those with single-ventricle CHD, specifically worries related to rescheduling surgeries or procedures, harm or death to the patient due to postponed care, and coming to the hospital or clinic for in-person care. Those who were post-heart transplant (mean 49.5 ± SD 27.2) were significantly more worried than all other disease groups (mean 27.8 ± SD 30.7; p < 0.0001) about obtaining medications during the pandemic. COVID-19 impact on personal finances was also most worrisome among the post-transplant patients (mean 62.7 ± SD 31.4) compared to all other disease groups (mean 52.3 ± SD 35.3; p = 0.04).

### Psychological stress and support needs

**Figure 2.**
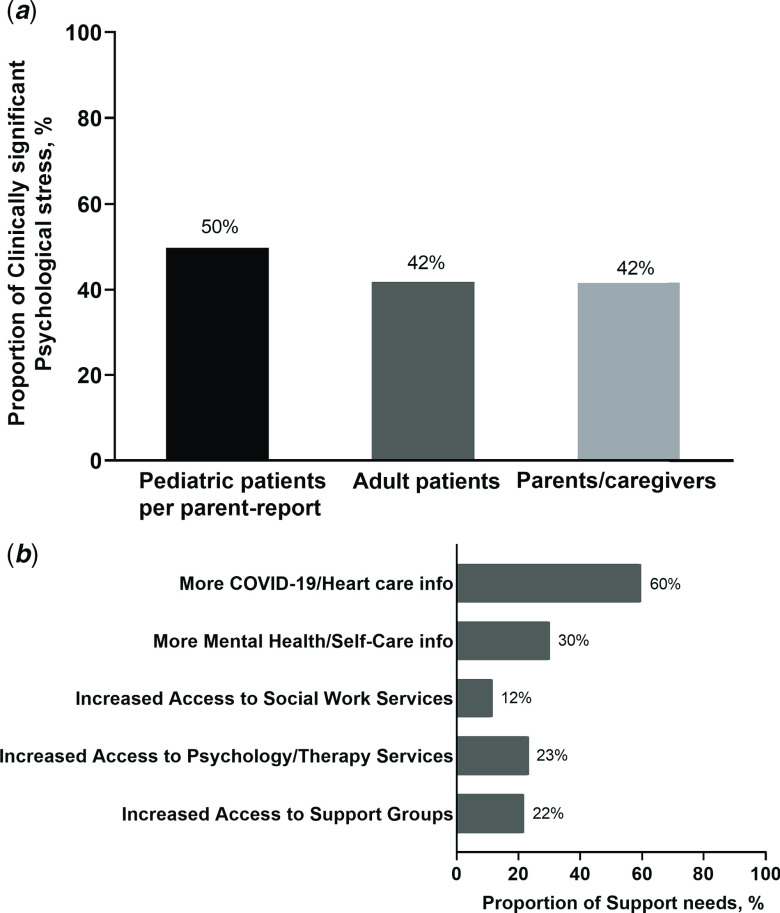
Psychological stress and support needs (n = 1220).

Overall, general psychological stress was high among the sample (Fig 2). Per parent report, 50% of children aged 5–17 years were experiencing clinically significant psychological stress as defined by a T-score >60 (i.e., >1 SD above the mean). Similarly, stress was high across adult CHD patients and parents/caregivers, with 42% with a T-score >60. There were no clinically significant differences in general psychological stress by CHD type across patients and caregivers; however, child stress as reported by parents was greatest among transplant recipients (mean 62.5 ± SD 6.3) compared to all other disease group (mean 59.1 ± SD 9.4; p = 0.03). Similar to regional differences observed in regard to COVID-19-related health concerns, patient and caregiver psychological stress was significantly higher in the Southern region of the United States of America (mean 59.3 ± SD 9.9 in South versus mean 56.4 ± SD 9.6 in other regions; p = 0.0003).

Participants reported on their support needs during the pandemic (Fig 2). Many (60%) stated that more information on COVID-19 and heart care would be helpful, while 30% felt additional information on managing mental health/stress would be helpful. More regular telemedicine check-ins with cardiology were desired by 28% of respondents. Nearly, a quarter (23%) of participants stated that increased access to psychology or therapy services would be helpful. Increased access to support groups (22%) and social work services (12%) were also desired.

## Discussion

The impact of the COVID-19 pandemic on the health and wellbeing of children and adults with acute and chronic non-COVID-19 illnesses has been tremendous. Healthcare system preparations for a surge in COVID-19 patients and considerable reductions in elective surgeries had notable impact on paediatric and adult CHD patients. Of those with a scheduled cardiac surgery or catheterisation, nearly 40% had already experienced postponed procedures in the early phase of the pandemic when this study was conducted. This is noteworthy as the study cohort reflected complex diseases, with a high proportion of single-ventricle CHD, transplant, and cardiomyopathy patients who are typically high utilisers of health resources.

The majority of patients and parents also endorsed considerable worry about COVID-19-related illness occurring in the patient with heart disease. Fear about returning to the hospital or clinic for in-person care during this early phase in the pandemic were great – at more than double the level of concern reported about harm to patient due to postponed care. Universally, results demonstrated clinically significant psychological stress in paediatric and adult patients with CHD, as well as their parents/caregivers.

Study findings highlight a number of necessary actions required of hospitals, healthcare providers, and heart centres. First, patients and caregivers desire more information about COVID-19 and their cardiac condition. As new research emerges about COVID-19 in patients with cardiac conditions, continued use of widely publicised webinars, articles, and fact sheets to quickly disseminate information will be important. Partnering with disease-specific support and advocacy groups is a meaningful vehicle for sharing such information, as the majority of participants reported getting their information from such sources.

It is also essential that patients and caregivers be provided with honest and up-to-date information specific to their safety and risks in regard to in-person healthcare. Direct communication with trusted healthcare team members is likely to be beneficial in helping patients and families navigate decision-making in terms of risk of exposure versus risk of postponed care. As data on hospital acquired COVID-19 infection becomes available and better understood, this information may be valuable to patients and families. A single-centre retrospective investigation in Wuhan, China, reported hospital acquired COVID-19 infection in 41% of 138 patients.^16^ Yet, in an early report of 121 healthcare providers with unprotected occupational COVID-19 exposure, only three tested positive for the virus in the 14 days following exposure.^17^ Thus, hospital-specific data will be important for patients and families returning to care.

Additionally, increased and continued use of telemedicine visits will be important, especially as most patients and caregivers in the current study felt that these visits adequately met their needs. Telemedicine visits provide opportunities for education, appropriate reassurances, and much needed support for concerned patients and caregivers.

Underscoring the considerable emotional toll the pandemic has had, an April 2020 study of psychological distress in the United States adults reported that 13.6% of the sample were experiencing serious psychological distress, which was greater than three times that observed in 2018.^18^ Although the current study used a different measure of stress which limits our direct comparison to these recent findings, rates of clinically significant psychological stress in our cardiac sample of patients and caregivers was very high, with 42–50% of patients or parent/caregivers endorsing high stress. Previous research has demonstrated that perceived psychological stress predicts risk for adverse physical health outcomes, increased depressive and anxiety symptoms, and increased substance use,^19^ underscoring the significance of our findings. Notably, the study recruitment period was prior to information about multisystem inflammatory syndrome in children being widely shared; thus, it is possible that the stress endorsed by many of the study participants is now an under-representation as COVID-19 risks to children may be greater than initially perceived.

Findings necessitate continued focus on the mental health of cardiac patients and their caregivers. A remarkable proportion of patients and families expressed interest in additional mental health and self-care information. Utilising support and advocacy groups to share evidence-based tips and resources is likely to be favourably received by patients and families. Possibly more important than ever, cardiac care should include mental health screening and referral for intervention for those in need of greater support. Whether in person or virtually, cardiac clinicians are encouraged to assess for mood, anxiety, stress, and coping in their patients. This can be quickly accomplished through the incorporation of visual analogue tools, such as the NCCN Distress Thermometer^20^ or Emotion Thermometers (http://www.psycho-oncology.info/ET.htm), which have demonstrated utility in cardiovascular settings.^21^

Referral to mental health colleagues should be provided for those with positive screens. Nearly, a quarter of study respondents indicated that they desired increased access to psychology or therapy services. This is noteworthy as previous research in adult CHD suggests that the majority of patients with mental health needs do not receive appropriate treatment.^9^ Options for telehealth-based mental health services, which are desired by this population,^22^ are growing rapidly.^23^ Increasing referral and access to such services for patients with emerging mental health concerns and those disconnected from previous supports because of the pandemic, such as the large proportion of children and adolescents who receive school-based mental health services,^24^ should be a priority. Lastly, as healthcare systems and hospitals face financial challenges, maintaining at least the current level of mental health support services for cardiac patients will be critical – both for identifying and intervening upon the distress patients and families are reporting, but also to address concerns related to returning to the hospital or clinic settings for care.

It is important to acknowledge the limitations of this survey study. To rapidly implement the survey, very minimal identifying/demographic data were collected, thus, limiting our ability to understand how factors such as race/ethnicity, sex, and socio-economic status may have impacted cardiac care disruptions and psychological stress. Our social media-based recruitment method unlikely sufficiently captured the views and experiences of underrepresented patients and families, including those of racial minority and lower socio-economic status. Given the considerable disproportionate rates of COVID-19 infection and mortality among African Americans in the United States of America,^25^ it is of high likelihood that cardiac care disruptions, COVID-19 health-related concerns, and psychological stress reported by our study participants are impacted by social determinants of health. It is critical that future research examines the role of demographic and social factors as it relates to paediatric and young adult cardiac care and psychological wellbeing during the pandemic, so that targeted interventions and support services can be deployed.

Additionally, due to our social media-based recruitment method, we do not know how many received the survey information but chose not to participate. It is likely that respondents were primarily those with personal internet access and those engaged in heart support or advocacy groups, thus, findings may not be generalisable to the diverse group of patients with heart disease and their families. For example, a large percentage of respondents represented the single-ventricle CHD community, which has a large social media presence. Applicability of findings to the broader CHD community should be cautiously considered. Lastly, the pandemic and society’s response to it has been dynamic and ever-evolving. Data captured in the current study were only a snapshot of experiences at the time, and longitudinal follow-up of cardiac care disruptions, health-related concerns, and psychological stress will be necessary.

In sum, this study of paediatric and adult CHD patients and caregivers demonstrated a considerable impact of the COVID-19 pandemic on cardiac care with nearly 40% or more of scheduled cardiac surgeries, catheterisations, and clinic visits postpone. Notable concerns about COVID-19 infection in CHD patients, considerable worries about returning to in-person healthcare, and high rates of clinically significant psychological stress were observed across the study participants in the early phase of the pandemic. Patients and caregivers with complex illness and high healthcare needs at baseline expressed greater concerns about COVID-19 infection than complications from delayed cardiac care. Further study of the impact of delays in care on clinical outcomes is needed. Promoting the physical health of patients with acute and chronic illness during the COVID-19 pandemic will require attending to their emotional needs.

## Supplementary material

For supplementary material accompanying this paper visit https://doi.org/10.1017/S1047951120004758. click here to view supplementary material
