# Supplementary material for: Impact of the COVID-19 pandemic on CHD care and emotional wellbeing
Source: Cardiol Young. 2020 Dec 14:1–7. doi: 10.1017/S1047951120004758 (PMC7783141; doi:10.1017/S1047951120004758)
Supplement: Supplementary file 1 [file S1047951120004758sup001.pdf]

Psychological Stress in Pediatric and Adult Congenital Heart Disease Patients and Caregivers During the COVID-19 Pandemic

The COVID-19 Pandemic has dramatically affected medical care throughout the world. Patients and caregivers of patients with congenital and other pediatric heart diseases have expressed worry about how COVID could affect them. Further, widespread changes in typical medical treatments and care delivery have occurred. Surgery, procedures, and clinic appointments have been reduced in efforts to stop the spread of the virus and preserve hospital resources for COVID-affected patients. With this survey we hope to better understand how care has changed across the world, how it has caused stress for patients and caregivers, and what specific issues have caused the most stress. We will ask a few questions about your/your child's or partner's specific heart disease, about specific changes in cardiology or cardiac surgery appointments and plans, specific concerns related to COVID-19, and some questions about your current level of stress. With this information, we hope to change our care practices during the pandemic to better serve our patients and better understand how to prioritize the backlog of care needs that will be present following the pandemic.

We invite you to participate in a survey research project to help learn more about stress related to cardiology care during the COVID-19 pandemic This brief, one-time survey should take less than 10 minutes to complete. We appreciate your participation.

Your participation is completely voluntary. Participation is not required. All information will be kept strictly confidential. No link from the survey to you/your child/your partner will be made. **The survey is anonymous**. The results of the survey will be kept indefinitely for use in future studies.

If you agree to complete the questionnaire, read the following statement of consent, and click on the "Start survey" button below which will send you to the web-based survey. Thank you for your help with this project.

I have read the information given above. I understand the meaning of this information. I consent to participate in the project. I am 18 years-old or older. I am completing this survey, and I am a person who has congenital or other pediatric heart disease, or I am the parent or caregiving partner of someone who has heart disease.

- ○ Start Survey
- ○ I do not Consent - Please close window

→

Please identify yourself

- ○ I am an individual with congenital or other pediatric heart disease
- ○ I am the parent or caregiver of an individual with congenital or other pediatric heart disease
- ○ I am the partner or spouse of > 1 year of an individual with congenital or other pediatric heart disease

→

What is your child's age in years?

Is your child currently admitted to a hospital?

No	Yes
O	O

Where is your child from?

- O United States of America
- O Other country

→

In which region of the USA does your child live?

- ○ Northeast
- ○ Midwest
- ○ South
- ○ West

My child has the following (check all that apply):

- ☐ Single ventricle congenital heart disease
- ☐ Two ventricle congenital heart disease
- ☐ Cardiomyopathy or disease of the heart muscle without structural heart disease
- ☐ Status post Heart Transplant

←　　→

Regarding single ventricle congenital heart disease, at what stage is your child?

- No operation yet
- s/p Stage 1 or shunt or Norwood operation
- s/p hybrid Norwood operation
- s/p Stage II or bidirectional Glenn or hemifontan operation
- s/p Fontan
- Other (write in)

Has your child had heart surgery in the past?

Yes	No
○	○

← →

Has your child had heart surgery in the past 6 months?

Yes	No
○	○

Has your child had more than 1 heart surgery in the past at any time?

Yes	No
○	○

← →

How many heart surgeries has your child had?

Did your child have a recent or upcoming surgery planned?

Yes	No
○	○

←

→

When was / is the surgery planned?

- March-July 2020
- July-Sept 2020
- October 2020-March 2021
- After March 2021

Has your child had a surgery date postponed due to the COVID pandemic?

Yes	No
○	○

← →

If your child has a surgery scheduled that has not been postponed, are you concerned about the potential impact of COVID pandemic on the surgery?

Yes	No
O	O

Did your child have a recent or upcoming cardiac catheterization planned?

Yes	No
O	O

← →

When was the cardiac catheterization planned?

- ○ March-July 2020
- ○ July-Sept 2020
- ○ October 2020-March 2021
- ○ After March 2021

Has your child had a cardiac catheterization postponed due to the COVID pandemic?

Yes	No
○	○

← →

If your child has a cardiac catheterization that has not been postponed, are you concerned about the potential impact of COVID pandemic on the procedure?

Yes	No
O	O

Did your child have a recent or upcoming cardiology clinic appointment planned?

Yes	No
O	O

← →

When was the upcoming cardiology clinic appointment?

- March-July 2020
- July-Sept 2020
- October 2020-March 2021
- After March 2021

Has your child had a clinic date postponed due to the COVID pandemic?

Yes	No
○	○

← →

If your child has a clinic date scheduled that has not been postponed, are you concerned about the potential impact of COVID pandemic on attendance in clinic?

Yes	No
○	○

Have you received information about rescheduling the appointment until after the pandemic?

Yes	No
○	○

← →

Has your child had an appointment changed from in-person to a video visit or phone call due to COVID-19?

Yes	No
O	O

← →

Did you feel the virtual visit was adequate to cover your needs?

Yes	No
○	○

← →

If you answered No, please explain why

Have you received general information about COVID-19 from your child's cardiologist/surgeon/center?

Yes No

Have you received information specific-to-you about COVID-19 from your child's cardiologist/surgeon/center?

Yes No

Have you received general information about COVID-19 from a heart disease support group, advocacy group, or social media group?

Yes No

← →

If you answered Yes, where did you receive this information? (check all that apply)

- ☐ Fact sheet
- ☐ Article
- ☐ Conference
- ☐ Webinar

← →

Please indicate which of the following concerns you as it relates to your child's heart care and health:

	Not concerned	A little concerned	Somewhat concerned	Moderately concerned	Very concerned
Obtaining medications during the pandemic	○	○	○	○	○
Not being seen in person by cardiology provider	○	○	○	○	○
Not being able to speak to or reach my cardiology provider	○	○	○	○	○
Postponed surgery or procedure	○	○	○	○	○
Postponed clinic visit	○	○	○	○	○
Challenges rescheduling surgery or procedure	○	○	○	○	○
Harm to your child due to postponed medical care	○	○	○	○	○
Death of your child due to postponed medical care	○	○	○	○	○
Coming to hospital or clinic for in-person care	○	○	○	○	○

How concerned are you about your child becoming ill from COVID-19?

- Not concerned
- A little concerned
- Somewhat concerned
- Moderately concerned
- Very concerned

How concerned are you about personally becoming ill from COVID-19?

- Not concerned
- A little concerned
- Somewhat concerned
- Moderately concerned
- Very concerned

How concerned are you about who will take care of your child if you get sick?

- Not concerned
- A little concerned
- Somewhat concerned
- Moderately concerned
- Very concerned

How concerned are you about the impact of COVID-19 on personal finances?

- Not concerned
- A little concerned
- Somewhat concerned
- Moderately concerned
- Very concerned

←

→

Please indicate how much each of the following your child has experienced since COVID-19 outbreak started

	None	Mild	Mild-Moderate	Moderate	Severe
Anxiety/Worry	O	O	O	O	O
Sadness	O	O	O	O	O
Anger	O	O	O	O	O
Changes in behavior (tantrums, attention problems, etc.)	O	O	O	O	O
Poor sleep	O	O	O	O	O
Difficulties taking medications	O	O	O	O	O

Please indicate which of the following would be helpful to your child to best support you during this stressful time and the months to follow (check all that apply):

- ☐ More regular video or phone check-ins with cardiology provider
- ☐ More information / resources on COVID-19 and heart care
- ☐ More information / resources on COVID-19 and managing mental health/stress
- ☐ Increased access to social work services
- ☐ Increased access to psychology / therapy services
- ☐ Increased access to support group
- ☐ Don't know / None of the above

← →

Perceived Stress (Ages 18+) -- Item Bank / Fixed Form

Please respond to each question or statement by selecting one option per row. Report on your own stress levels, not those of your child.

In the past month...

	Never	Almost Never	Sometimes	Fairly Often	Very Often
How often have you been upset because of something that happened unexpectedly?	O	O	O	O	O
How often have you felt that you were unable to control the important things in your life?	O	O	O	O	O
How often have you felt nervous and “stressed”?	O	O	O	O	O
How often have you felt confident about your ability to handle your personal problems?	O	O	O	O	O
How often have you felt that things were going your way?	O	O	O	O	O

	Never	Almost Never	Sometimes	Fairly Often	Very Often
How often have you found that you could not cope with all the things that you had to do?	○	○	○	○	○
How often have you been able to control irritations in your life?	○	○	○	○	○
How often have you felt that you were on top of things?	○	○	○	○	○
How often have you been angered because of things that happened that were outside of your control?	○	○	○	○	○
How often have you felt difficulties were piling up so high that you could not overcome them?	○	○	○	○	○

← →

PROMIS Parent Proxy Item Bank v1.0 - Psychological Stress Experiences - Short Form 8a

Psychological Stress Experiences - Short Form 8a

Please respond to each question or statement by selecting one option per row.

In the past 7 days...

	Never	Rarely	Sometimes	Often	Always
My child felt stressed	O	O	O	O	O
My child felt that his/her problems kept piling up	O	O	O	O	O
My child felt overwhelmed	O	O	O	O	O
My child felt unable to manage things in his/her life	O	O	O	O	O
Everything bothered my child	O	O	O	O	O
My child felt under pressure	O	O	O	O	O
My child had trouble concentrating	O	O	O	O	O
My child felt he/she had too much going on	O	O	O	O	O

→

Thank you for completing this survey.

If you or your child or partner are experiencing a mental health emergency, please contact 911 or your local emergency department immediately. You may also utilize the National Suicide Prevention Line for any additional mental health emergencies at 1-800 273-8255 or the Crisis Text Line by texting “HOME” to 741741.

←

→
